# A fuzzy intelligent system to assess midwives’ burnout conditions

**DOI:** 10.18332/ejm/143363

**Published:** 2021-02-14

**Authors:** Stavroula Barbounaki, Victoria G. Vivilaki

**Affiliations:** 1 Department of Midwifery, School of Health and Care Sciences, University of West Attica, Athens, Greece

**Keywords:** fuzzy intelligent system, burnout, midwifery burnout, fuzzy logic

## Abstract

**INTRODUCTION:**

Midwives’ burnout affects their effectiveness and the quality of the services they provide to pregnant women as well as the quality of the collaboration with medical staff. The burnout depends on a number of factors that can exhibit high variability over time. This creates the necessity of introducing intelligent approaches that assess changes in behavior, environmental factors, working conditions, and to make decisions to optimize the physical and mental health of midwives. The aim of this study was to employ fuzzy logic to design a Fuzzy Intelligent or Inference System (FIS) that assesses midwives’ burnout level by emulating the reasoning of human experts.

**METHODS:**

The proposed FIS addresses the assessment of midwives’ burnout comprehensively since it incorporates findings following a thorough analysis of the relevant literature, as well as assimilates experts’ knowledge elicited through semi-structured interviews. Additionally, fuzzy rules are more intuitive and thus easier to understand and modify by human users than dealing and translating numerical results. The FIS performance is compared and evaluated against experienced midwives.

**RESULTS:**

Findings confirm the ability of the proposed FIS to produce judgments that are closer to experts’ consensus, as expressed by their aggregated assessment.

**CONCLUSIONS:**

The proposed FIS is evaluated by comparing its results with judgments made by experts, suggesting that fuzzy logic allows precise and personalized assessment of midwives’ burnout levels. The proposed FIS can be used to evaluate burnout, support organizations to develop burnout policies as well as used as a research instrument to investigate interrelationships of burnout factors.

## INTRODUCTION

Burnout related research is increasing rapidly these days because of the stressful nature of midwifery. Burnout is a syndrome possessing three main characteristics. The first refers to emotional exhaustion. Emotional exhaustion occurs in cases where professionals feel as if their emotional strength is being depleted. As a result, they do not effectively engage in their work, as their ability to respond to the needs of their patients is decreasing^1,2^. The second characteristic refers to depersonalization, namely the distance professionals subconsciously take sometimes from their service recipients and the development of a cynical attitude towards them^1,2^. The third characteristic refers to reduced personal accomplishment, suggesting that people who suffer from the burnout syndrome have a decreased ability to evaluate themselves positively with regard to their work performance^2,3^. Midwives are faced with a lot of emotional demands daily, as they are called to provide support in a very stressful period of a woman’s life^2,4-7^. Even more so, if there are any complications or difficulties in childbirth, stress increases and midwives are at risk of vicarious secondary traumas^7-9^. Meanwhile organizational and professional factors (modifiable or non-modifiable) may also increase stress levels, making the working environment intolerable. Such factors include a heavy workload, staff shortages, the shift system, bullying, and lack of high-quality managerial support^2,10-13^.

As burnout cases in midwives further increase, it becomes evident that there is a pressing need to address the factors that may influence/lead to the development of this syndrome^2,14-16^. In an attempt to address this issue, Maslach et al.^1^ stated that each midwife carries some distinctive qualities to the work (age, years in profession, coping styles, social support), qualities which may later on play a huge part in the risk of experiencing burnout. For instance, research showed that younger nurses are more susceptible to burnout, as they have not yet developed the necessary skills to deal with stressful situations^1,17-19^. Further research on the factors influencing burnout could eventually benefit healthcare institutions, by tackling the issue of midwife shortage^16^.

Bearing this in mind, efforts have been made internationally to gain knowledge on the burnout syndrome. More specifically, studies have been performed in the UK^20,21^, Denmark^22,23^, Australia^15^, Japan^24^, Ireland^25^, Sweden^26^, Norway^27^, Lithuania^28^, Jordan^29^ and Canada^30^.

As burnout studies continue to develop, two main measurement scales seem to prevail, as they best assist in exploring this multidimensional syndrome. The Maslach Burnout Inventory (MBI), one of the dominant measurement scales, emphasizes the experiences of people working in the human services and includes symptoms relevant to exhaustion, but also depersonalization and reduced personal accomplishment^3,31^, as opposed to some researchers’ reservations regarding the causal relationship between the two final domains and burnout^23,32^. Furthermore, MBI implies that the emotional load of working with clients may also relate to high burnout levels. However, it should be noted that the specific scale is only commercially available^1,3,31^ and does not clarify whether it reflects on the syndrome as a state, a coping strategy or simply an effect^31,32^. In contrast to MBI, the CBI (Copenhagen Burnout Inventory) created by Kristensen et al.^32^ does not include the concepts of depersonalization and reduced personal accomplishment. On the other hand, this tool consists of three sections, namely personal, work-related and client-related, which refer to the source or causality of physical and emotional fatigue rather than the symptoms^23,27,31,33-38^. Winwood and Winefield^38^ compared the two measurement tools, and found the CBI to be superior, as it provides a precise definition of burnout as a fatigue phenomenon, it presents high reliability and validity, it separates work and personal factors, and it is suitable for healthcare services because of the inclusion of the client-related domain^1,32,39,40^.

### Factors affecting midwives’ burnout

Thorough literature analysis reveals that there are some factors that are repeatedly reported by midwives in most of the countries, as probable causes of burnout. In their study, Sidhu et al.^41^ collected and analyzed those factors. The most common relate to insufficient organizational support, stressful working environment^27,30,34,35,42-44^, working in non-case load/non-continuity models of care (i.e. shifts in hospitals)^33,34,37,45-47^, less midwifery experience^2,22,35,36,43^, young age^27,34-36,43^, high workload combined with non-existent time-off^6,30,34,35,43^, traumas^48-50^, and conflicts with colleagues/low recognition^34,35,42,50^. Other less frequent factors that were reported concerned low job satisfaction^50,51^, lack of support by family/friends^43-45^, low pay^36,43,50^, and no children^31,35,46^. Meanwhile, there were a few studies that included factors such as low job autonomy^6,49^, serving clients with diverse psychosocial needs^2,43^, seniority^34,50^, being married^51,52^, worrying about own health^35,51^, depression^30,31^, having young children^30,43^ , and being single^27,43^. Finally, factors that were reported in at least one study were: low exercise, working night shifts^2^, lower percentage of attended home births, passive coping style^22^, insufficient education^51^, and lack of career opportunities^34^. Also, further training on efficient team work and communication skills is necessary among community midwives, in order for them to be able to share the excessive workload^6^. On the other hand, caseload midwives reported lower levels of burnout, irrespective of the excessive working hours, due to the continuity and independence that this model provides^6^.

Future research on the causality and experience of burnout in midwifery should further advance, as midwives’ mental health is imperative for providing high-quality maternal care^27,36,41^. For example, researchers could develop mixed methods, but also perform cross-cultural studies between countries with different care models^36^, in order to examine aspects of the syndrome^31^. This study suggests the development of a fuzzy system that incorporates available knowledge, expertise as well as the intuition of expert midwives, in order to assess burnout conditions in a comprehensive manner. The proposed FIS can assist midwives and organizations to derive policies for addressing burnout as well as used as a research tool to investigate additional factors and their implications. To our best knowledge this is the first fuzzy inference system for assessing midwives’ burnout.

## METHODS

### Fuzzy intelligent system

Fuzzy intelligent systems design-methodologies are well documented^53^. They include the steps that are illustrated below:

Step1: Identify one or more input, as well as one or more output, linguistic variables.

Step2: Define the fuzzy sets for each of the variables.

Step3: Specify the fuzzy rules that associate fuzzy input variables to fuzzy output variables.

The methodology steps followed for the design and development of the proposed FIS are explained in the sections below.

### Step 1: Selection of the input and output linguistic variables

There are many system parameters that can be monitored and that can be used as input to assess the burnout conditions of midwives and subsequently to derive the necessary policies in order to address any side-effects. Following thorough analysis of the relevant literature, our approach considers as input the following variables: education, organizational factors, working conditions, interpersonal relations, experience, individual factors, and family support. The single output is the variable level-of-burnout that shows the extent midwives are exposed to burnout.

### Step 2: Defining the fuzzy sets for each of the variables

**Figure 1:**
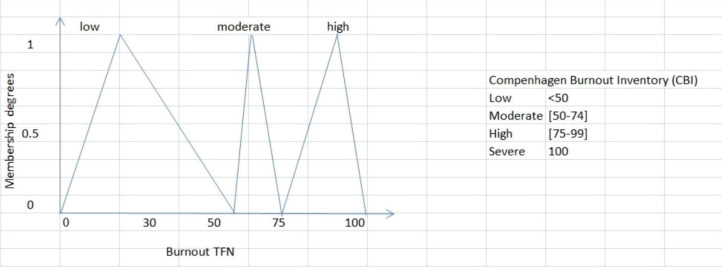
Triangular fuzzy numbers (tfn) for burnout level

Each linguistic variable is associated with a membership function, which maps elements from the variable’s universe of discourse to a value within the 0–1 interval. This study uses triangular fuzzy sets, due to their solid theoretical basis and simplicity^54^. The membership function of triangular fuzzy set *(a, m, b)* can be calculated according to the following equation^55^:

$$f_{A}\left(x\right)=\{\begin{array}{ccc} \frac{x-a}{m-a}, & a\leq x<m, & m\neq a\\ \frac{b-x}{b-m}, & m\leq x<b, & m\neq b\\ 0, & otherwise & \end{array}$$ (1)

where *a, m, b* are real numbers. The triangular fuzzy sets which are shown in Figure 1, for burnout level, are defined drawing on the Copenhagen Burnout Inventory (CBI) scoring^31^.

The input variables’ fuzzy sets used are shown in Table 1. Membership functions were manually checked and calibrated, through several tests, to ensure the system responded accordingly.

**Table 1:** Input variables fuzzy sets’ membership thresholds

**Education level (EL)**	
Low	(0 1.5 4)
High	(4.5 7 10)
**Organizational factors (O)**	
Low	(0 1.5 4)
High	(4.5 7 10)
**Working conditions (W)**	
Low	(0 1.5 4)
High	(4.5 7 10)
**Interpersonal relations (IR)**	
Low	(0 1.5 4)
High	(4.5 7 10)
**Experience (E)**	
Low	(0 1.5 4)
High	(4.5 7 10)
**Individual factors (I)**	
Low	(0 1.5 4)
High	(4.5 7 10)
**Family support (F)**	
Low	(0 1.5 4)
High	(4.5 7 10)

### Step 3: Defining the fuzzy rules that associate fuzzy inputs to fuzzy outputs

Linguistic rules are expressed in a form such as ‘If premise then consequent’, where premises represent the FIS input variables and consequents are associated with the fuzzy intelligent system outputs. The number of the FIS inputs and outputs designates the upper limit on the number of elements in the premises and consequents. The rules (strategies) on how to assess and manage burnout were derived empirically by studying the relevant literature. Deriving strategies to manage midwives’ burnout is more an art than a science, and there are rules-of-thumb.

*Rules base set*

For any combination of 4 of the 7 input variables that are Low, the burnout is taken as High.

For example:

*If [EL is Low] AND [O is Low] AND [W is Low] AND [IR is Low] then [burnout is High].*

Where the input variables are denoted by: EL: education level. O: organizational factors. W: working conditions. IR: interpersonal relations. E: experience. I: individual factors. F: family support (Table 1).

This results in 35 fuzzy rules, using the combinations equation:


$${}_{4}^{7}C=7!/[4!(7-4)!]$$

where 4! = 4×3×2×1. These 35 fuzzy rules form the *rules base set*, which is manually checked for consistency and completeness.

### Fuzzy reasoning with the Mamdani Min-Max approach

The Mamdani method of fuzzy inference draws on the Min-Max implication function. The operators AND and OR are used to associate the antecedents and specify the result after firing each rule. The Max function is used to aggregate the rules. It is also known as Min–Max rule or the correlation-minimum implication^56^. The Mamdani method assumes a set of (*r*) disjunctive rules. Each rule is an *n*-input single output rule such as:


$$IF\;x_{1}={\tilde{A}}_{1}^{k}\;AND\;x_{2}={\tilde{A}}_{2}^{k}\;AND\;x_{i}={\tilde{A}}_{i}^{k}\;THEN\;y={\tilde{B}}^{k}$$

where $A_{1}^{k}$,..., $A_{i}^{k}$ *and B^k^* are fuzzy sets

with

*k* = 1,..., *r* representing the rules,

*x_i_* , $A_{i}^{k}$ are the antecedents of the k-the rule, where *x_i_* , $A_{i}^{k}$ are the input and the fuzzy set respectively, with

*i* =1,..., *n* inputs representing the rules’ *n*-inputs, and

*y*, ${\tilde{B}}^{k}$ representing the single consequent of the *k*-th rule.

Let us assume the inputs $\left(x_{1}=x_{1}^{'},x_{2}=x_{2}^{'},...,x_{n}=x_{n}^{'}\right)$. Following Mamdani’s inference, the implication of the first rule is calculated using the formula:

$$\mu_{{\tilde{B}}^{1}}\left(y\right)=\min\left(\mu_{{\tilde{A}}_{1}^{1}}\left(x_{1}^{'}\right)\mu_{{\tilde{A}}_{2}^{1}}\left(x_{2}^{'}\right),...,\mu_{{\tilde{A}}_{n}^{1}}x_{n}^{'}\right)$$ (2)

The aggregation of all (*r*) rules is calculated using the formula:

$$\mu_{\tilde{B}}\left(y\right)=\max\left\{\;\mu_{{\tilde{B}}^{1}},\;\mu_{{\tilde{B}}^{2}},...,\mu_{{\tilde{B}}^{r}}\right\}$$ (3)

The rules aggregation results in a fuzzy set, which is the conclusion after firing the rules in the knowledge base.

## RESULTS

### Architecture of the Fuzzy Intelligent System (FIS)

**Figure 2:**
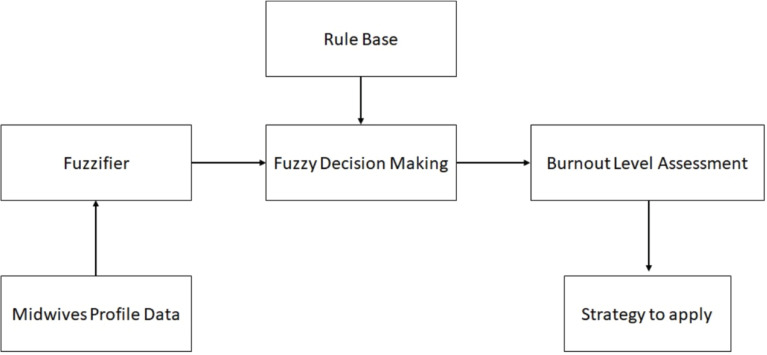
Logical architecture of the Fuzzy Intelligent Systems to assess a midwives’ burnout

The logical architecture of the FIS is shown in Figure 2. The FIS is a multiple input/single output type of system that accepts as input data related to the midwives’ burnout level determinants, and produces as output the assessment of burnout level for each midwife. The functionality of the system components is as follows:

- The fuzzifier calculates the value of the input variables. Finally, it converts input data into suitable fuzzy values, according to the fuzzy sets shown in Table 1.
- The *rules base set* comprises the 35 fuzzy rules, as defined above.
- The fuzzy decision-making component executes the fuzzy rules for a given set of inputs, by adopting the Mamdani approach.

The decision-making component of the FIS was implemented in MATLAB. The rules are defined in the system as shown in Supplementary file Figure 1.

The relationships between the inputs used by the fuzzy intelligent system and the single output, i.e. burnout level of midwives, are shown in Supplementary file Figure 2.

Supplementary file Figure 2 provides an overview of the FIS, using MATLAB’s surface viewer and shows the behavior of the input variables used in the proposed FIS and the estimated output. Supplementary file Figure 2 shows that a moderate level of experience combined with poor working conditions results in high levels of burnout.

### Fuzzy intelligent system evaluation

Evaluating and validating intelligent systems is a major issue. In order to evaluate the proposed FIS, a number of test cases were examined. The burnout assessments produced by the FIS were compared with evaluations made by a group of 15 expert midwives. Input data representing the profile of a midwife is read by the FIS and the burnout level is assessed. The following example assumes: EL=low, O=moderate, W=low, IR=high, E=low, I=low and F=moderate. The FIS returns a quantitative Burnout level=0.696. By applying Formula (1), the result is fuzzified thus returning the FIS qualitative assessment of Burnout=moderate. Supplementary file Figure 3 shows a sample of the system output for this particular test case.

**Figure 3:**
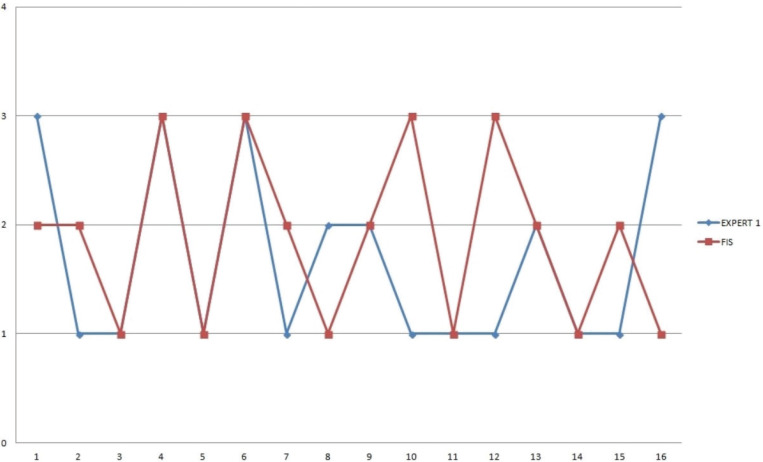
Comparison of the proposed FIS and Expert-1 assessment

The expert midwives were given the same test data to judge and present their assessment, in terms of the burnout TFNs. Experts’ judgments (e_i_), however, do not always agree on the burnout level. An aggregation of the Expert’s judgments is therefore needed. It is usually calculated by calculating the geometric mean, which is assumed to capture expert consensus more accurately^57,58^. This study uses the TFNs with geometric means to represent expert consensus. Thus, the aggregated TFN of the obstetricians’ responses is denoted simply as a triple, *e_agg_(a, m, b)*, where:

*a = min(e_i_)* (4)

represents the lowest of all experts’ judgment, and *i* = 1,...,*n* represent the number of obstetricians, (*e_i_*) represents the response of the i-th obstetrician and

$$\text{m}=\sqrt[{\text{n}}]{\Pi_{\text{i=1}}^{\text{n}}\;{\text{e}}_{\text{i}}}$$ (5)

the geometric mean of the (e_i_) indicating the aggregation of all experts’ judgments, and

*b* = max (*e_i_*) (6)

represents the highest of all the experts’ judgments.

The aggregated diagnosis is subsequently fuzzified using Formula (1), thus expressing the experts’ judgments in terms of low, moderate or high level of burnout.

Since the proposed FIS accumulates knowledge acquired by experts as well as comprehensively associates variables that affect burnout, the FIS assessment is compared against the aggregated experts’ judgment. A sample data of 16 midwife profiles was used to evaluate the FIS. The chart in Figure 3 shows the proposed FIS and Expert-1 assessments. The FIS results agree with the Expert-1 judgment in 8 cases.

**Figure 4:**
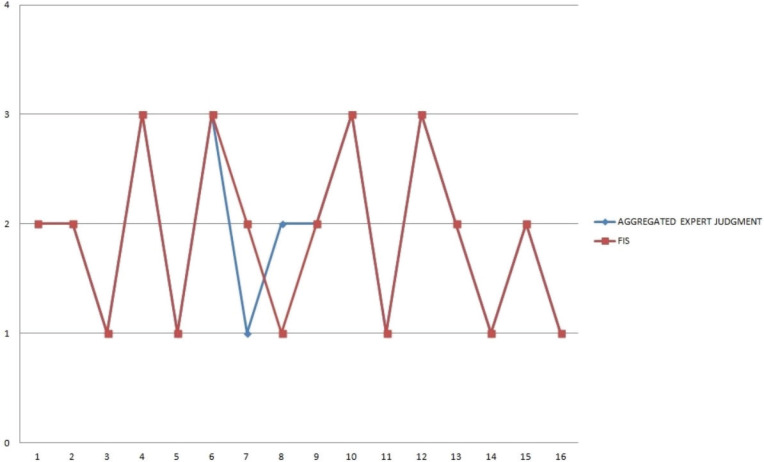
Comparison of the proposed FIS and expert consensus

The chart in Figure 4 shows the proposed FIS and experts’ aggregated assessments. The graph indicates that FIS results are closer to the experts’ consensus than some of the individual experts.

Thus, FIS benefits from accumulating knowledge, returning assessment that would be accepted by the majority of the human experts. FIS results and experts’ consensus judgments agree in 14 cases of the 16 midwife profiles examined.

## DISCUSSION

Being aware of the factors influencing burnout, could eventually give organizations the opportunity to develop proper strategies in order to decrease stress and prevent burnout occurrence (i.e. organize clinical supervision sessions, provide subsidized fees to promote physical exercise, improve communication, team cohesion and interaction between healthcare professionals through sessions etc.)^2,30^. Research studies indicate the effectiveness of the CBI and the MBI scales. However, this study proposes the development of an FIS that not only adopts a comprehensive perspective of assessing the burnout but it also incorporates experts’ knowledge, experience, and intuition.

## CONCLUSIONS

The proposed FIS is evaluated by comparing its results with judgments made by experts, indicating that the use of fuzzy logic allows for precise and personalized assessment of midwives’ burnout levels. The proposed FIS can be used as a tool to evaluate burnout, as a tool for organizations to examine policies when dealing with burnout, as well as a research instrument to further investigate the factors that affect burnout.

## Data Availability

Data sharing is not applicable to this article as no new data were created.
